# Association of EGFR gene polymorphisms rs2072454 and rs2227983 with lung cancer susceptibility in a Western Algerian population: a case-control study

**DOI:** 10.1186/s43046-026-00364-9

**Published:** 2026-05-11

**Authors:** Linda Temimi, Noria Bouras, Abdelkader Bousahba, Ahlem Megaïz, Malika Lechar, Meriem Mekedem, Sonia Seddiki, Tewfik Sahraoui

**Affiliations:** 1 Department of Biology, Faculty of Natural and Life Sciences, Laboratory of Biology of Development and Differentiation (LBDD), University of Oran 1 Ahmed Ben Bella, Oran, Algeria; 2 Department of Applied Molecular Genetics, Faculty of Natural and Life Sciences, University of Science and Technology of Oran, Mohamed Boudiaf “USTO-MB”, Oran, Algeria; 3 Department of Medical Oncology, University Hospital Center of Oran, Oran, Algeria; 4 Department of Medicine, Faculty of Medecine, University of Oran 1 Ahmed Ben Bella, Oran, Algeria; 5 Department of Biology, Faculty of Natural and Life Sciences, Laboratory of Biology of Microorganisms and Biotechnology (LBMB), University of Oran 1 Ahmed Ben Bella, Oran, Algeria

**Keywords:** Lung adenocarcinoma, *EGFR*, Single nucleotide polymorphism, Genetic susceptibility, PCR-RFLP

## Abstract

**Background:**

Data on specific single nucleotide polymorphisms (SNPs), including rs2072454 and rs2227983, remain limited, particularly in North African populations. This study aimed to evaluate the association between these two SNPs and lung cancer risk in a Western Algerian population.

**Methods:**

This is a case-control study including 143 participants, 73 lung cancer patients recruited from the University Hospital Centre of Oran, and 70 healthy controls recruited from the blood transfusion centre of the University Hospital Establishment of Oran (UHEO) and volunteer pool. Genotyping of *EGFR* rs2072454 and rs2227983 polymorphisms was performed using polymerase chain reaction- restriction fragment length polymorphism (PCR-RFLP). Statistical analyses were conducted using R software, with logistic regression models adjusted for gender and smoking status.

**Results:**

The CT genotype of rs2072454 showed a nominal association with increased lung cancer risk under the overdominant model (OR = 2.35, 95% CI: 1.04–5.30, *p* = 0.04). For rs2227983, while the dominant model (AG/ GG vs AA) demonstrated the best fit based on AIC/BIC criteria, however, only the AG genotype showed a borderline association in adenocarcinoma cases under the codominant model (OR = 2.76, 95% CI: 1.0-7.5, *p* = 0.04). No significant haplotype associations or linkage disequilibrium was observed between the two SNPs.

**Conclusions:**

These findings suggest potential, but uncertain, associations between EGFR polymorphisms and lung cancer susceptibility in this population. The results should be interpreted cautiously due to the limited sample size and require validation in larger cohorts.

## Background

 Worldwide, lung cancer ranks first in terms of incidence with almost 2.5 million new cancer cases and the leading cause of cancer-related deaths with more than 1.8 million deaths (18%), in 2022 [1]. Non-small cell lung cancer (NSCLC) is the most frequent form of lung cancer with 80–85%. Most of these patients are diagnosed at an advanced, unresectable disease [2]. The molecular heterogeneity of lung cancer has allowed for the development of targeted therapies. Multiple genetic abnormalities in chromosomes, oncogenes, and tumour suppressor genes have been identified, leading to advances in these treatments [3].

The epidermal growth factor receptor (EGFR) is a transmembrane protein and a member of the ErbB family of receptor tyrosine kinases. It mediates signalling pathways that regulate growth, survival, proliferation, and differentiation in mammalian cells [4]. The *EGFR* gene, which encodes this protein, is located on chromosome 7p11.2 and consists of 244,589 base pairs, containing 28 exons and 27 introns [5].

Given *EGFR*’s pivotal role in oncogenesis, there is growing interest in understanding the implications of *EGFR* polymorphisms, particularly single nucleotide polymorphisms (SNPs), that may influence an individual’s risk of developing cancer. These SNPs are of special relevance in lung cancer, where EGFR signalling is frequently dysregulated. However, there are limited correlative studies linking specific EGFR SNPs (e.g., rs2072454 and rs2227983) to lung cancer risk remain limited, especially in understudied populations.

In this study, we examined the association between the two SNPs rs2072454 and rs2227983 located in the *EGFR* gene and lung cancer risk in a Western Algerian population representing a North African population.

The rs2072454 polymorphism is located in exon 4 of the *EGFR* gene, within two bases of an exon–intron junction and corresponds to the ligand-binding domain of the protein [6, 7].

The rs2227983 polymorphism, previously known as rs11543848, is located in exon 13 of the *EGFR* gene. It is often referred to as the R521K polymorphism, as it results in the substitution of arginine with lysine at codon 521, on the border between extracellular subdomains III and IV of the protein. This polymorphism is expressed in the extracellular domain, which controls the binding arm [8–11].

Given limited data on *EGFR* SNPs in North African populations, the present study was conceived as a hypothesis-generating effort aimed at assessing the association between lung cancer and the EGFR rs2072454 and rs2227983 SNPs identifying potential genetic risk factors in a North African population.

## Methods

### Participants

This case-control study included 73 cases with confirmed diagnosis of primary lung cancer and 70 healthy controls with no history of cancer. Lung cancer patients were recruited prospectively between June and August 2024 from the Medical Oncology Department of the University Hospital Centre of Oran (UHCO). Only treatment-naïve patients were included to avoid confounding effects of therapy, regardless of the type or stage of cancer. 

Healthy controls were recruited from the blood transfusion centre of the University Hospital Establishment of Oran (UHEO) and supplemented by volunteers.

In this study, we prioritized real-world clinical recruitment over strict matching, which may affect generalizability but reflects the population’s epidemiological profile.

Histological types and stages data of the patients were collected from their medical records. Demographics and smoking status of both cases and controls were collected using a questionnaire.

Given the exploratory nature of this study, the sample size was not determined by formal power calculations but was based on feasibility and available patient data.

All participants received a full explanation of the study objectives and procedures and provided informed consent, and the study was approved by the relevant institutional ethics committees.

### Sampling

Blood sample of each participant was collected in a 4 ml ethylene diamine tetra acetic acid (EDTA) tube and stored at -20 °C for DNA extraction and genotyping.

### Genomic DNA extraction

DNA was extracted from whole blood using QIAamp DNA Blood Mini Kit (Lot n°175023480, GERMANY). The extraction protocol was performed according to the manufacturer’s instructions. The yield of DNA was assessed using Qubit 2.0 fluorometer (REF Q32866, Singapore). For further use, the extracted DNA was stored at -20 °C.

### Genotyping

Information about the SNPs were obtained from the dbSNP (RRID: SCR_002338). The two *EGFR* SNPs rs2227983 and rs2072454 were amplified by PCR (Polymerase Chain Reaction) and genotyped by RFLP (Restriction Fragment Length Polymorphism) methods.

The primers’ sequences used for PCR amplification were previously described along with the PCR conditions and product size [6].

The rs2227983 and rs2072454 SNPs were genotyped respectively using the BstN1 (New England Biolabs) and the Bstu1 (New England Biolabs) restriction enzymes and digestion reaction conditions were performed according to the manufacturer’s instructions.

The *EGFR* rs2072454 SNP was categorized as digested (CC homozygote), non-digested (TT homozygote) and partially digested (CT heterozygote). Similarly, the *EGFR* rs2227983 SNP was categorized as digested (GG homozygote), non-digested (AA homozygote) and partially digested (AG heterozygote).

The size of the PCR products and the restriction profiles of the amplicons were verified by electrophoresis using respectively 2.5% and 3% of agarose gel stained with ethidium bromide. The electrophoresis gel was visualised under UV light using a gel documentation system (Gel Doc N-90 K, Wiesloch, GERMANY).

### Statistical analysis

All statistical analyses were conducted using the R software. Categorical variables are reported as percentages, and continuous variables are reported as mean ± standard deviation. 

The haplotype construction along with linkage disequilibrium (LD) calculation were performed using the R software. The association between the genetic polymorphisms and lung cancer risk along with the haplotype analysis were assessed through multivariable logistic regression models adjusting for gender and smoking status. Odds ratios (ORs) with 95% confidence intervals (CIs) were reported. 

To identify the optimal genetic inheritance model (codominant, dominant, recessive, overdominant, or additive) for each SNP, we compared models using Akaike information criterion (AIC) and Bayesian information criterion (BIC), which balance model fit and complexity. The model with the lowest AIC/BIC values was selected as optimal.

Given the exploratory nature of the study and multiple model testing, p-values should be interpreted cautiously. 

## Results

The study included 143 subjects, 73 lung cancer patients and 70 healthy controls. The group of patients was predominantly male (98.6%) and consisted mainly of smokers (95.9%), with a mean age of 61 ± 10.7. In contrast, the control group included 84.3% males, with 51.4% smokers and a mean age of 57.3 ± 10.3 years. 

Among patients, 74% had adenocarcinoma (ADC), 22% had squamous cell carcinoma (SqCC) and 4% had small cell lung cancer (SCLC). All the patients were diagnosed either with a stage III or IV of the disease respectively at 32 and 68%.

### Genotypic and allelic frequencies of the two SNPs (rs2072454 and rs2227983) in both controls and lung cancer cases

The frequency distribution of the gender, smoking status, genotypes and alleles in both controls and lung cancer groups are shown in Table 1. The CT heterozygous genotype of the rs2072454 was the most frequent in both groups. Moreover, regarding the rs2227983, the AG heterozygous genotype was the most frequent in the patients’ group while in the control group, the most frequent was the AA homozygous genotype.

**Table 1:** Frequency distribution of the *EGFR* SNPs (rs2072454 and rs2227983) genotypes and alleles adjusted for gender and smoking status among lung cancer patients’ group, lung adenocarcinoma patients’ subgroup and control group

	Controls *N* = 70 (%)	Lung Cancer Patients *N* = 73(%)	OR (95% CI)	*p*-value	Lung ADC Patients *N* = 54 (%)	OR (95% CI)	*p*-value
rs2072454							
Gender							
Male	59(84.3)	72(98.6)	7.88[0.8–81.6]	0.08	53(98.1)	2.55[0.4–15.5]	0.3
Female	11(15.7)	1(1.4)	-	-	1(1.9)	-	-
Smoking Status							
Smoker	36(51.4)	70(95.9)	22.8[6.2–83.9]	2.4e^− 6^	51(94.4)	17.1[4.6–62.6]	1.9e^− 5^
Non-smoker	34(48.6)	3(4.1)	-	-	3(5.6)	-	-
CT	37 (52.9)	43 (58.9)	2.35[1.04–5.30]	0.04*	31 (57.4)	2.02[0.9–4.67]	0.10
TT/CC	33 (47.2)	30 (50)	1	-	23 (43.6)	1	-
Allele frequencies							
Allele C	0.5 (50)	0.5 (50)	1	1	0.5 (50)	1	1
Allele T	0.5 (50)	0.5 (50)	1	-	0.5 (50)	1	-
rs2227983							
Gender							
Male	59(84.3)	72(98.6)	6.16[0.6–64.6]	0.12	53(98.1)	2.42[0.4–15.3]	0.35
Female	11(15.7)	1(1.4)	-	-	1(1.9)	-	-
Smoking Status							
Smoker	36(51.4)	70(95.9)	16.8[4.7–59.7]	1.4e^− 5^	51(94.4)	12.8[3.6–45.8]	9.4 e^− 5^
Non-smoker	34(48.6)	3(4.1)	-	-	3(5.6)	-	-
AG	17 (24.3)	34 (46.6)	2.43[0.95–6.19]	0.06	24 (44.4)	2.76[1.01–7.6]	0.04**
GG	21 (30)	20 (27.4)	1.32[0.5–3.48]	0.57	18 (33.3)	1.88[0.7–5.3]	0.22
AA	32 (45.7)	19 (26)	1	1	12 (22.2)	1	-
Allele frequencies							
Allele A	0.57	0.49	1	0.96	0.45 (45)	1	0.91
Allele G	0.42	0.51	1.41[0.7–2.56]	-	0.55 (55)	1.65[0.9–3.02]	-

ORs and 95% CIs are derived from logistic regression adjusted for gender and smoking status

*ADC* Adenocarcinoma, *OR* Odds Ratio, *CI* Confidence Interval

* Significant association with lung cancer risk

** Significant association with lung adenocarcinoma risk

Across all logistic regression models (for both rs2072454 and rs2227983, and in both the overall lung cancer group and adenocarcinoma subgroup), smoking status maintained a robust association with disease risk (*p* < 0.05), while gender consistently showed a non-significant trend (*p* > 0.05). After adjusting for these covariates, the heterozygous CT genotype of rs2072454 showed a nominal association with increased lung cancer risk under the overdominant model (CT vs. CC/TT) (OR = 2.35, 95% CI: 1.04–5.30, *p* = 0.04, AIC = 157.6, BIC = 169.4), suggesting this genotype may confer elevated susceptibility independent of gender or smoking status as individuals carrying the CT heterozygous genotype may have higher lung cancer risk compared to either homozygous genotype (CC or TT). In contrast, no significant association was observed for rs2227983 in the overall analysis (*p* > 0.05). However, the overdominant model (AA/GG vs. AG) showed the lowest AIC (158.6) and BIC (170.4), suggesting it may best represent the underlying inheritance pattern despite lacking individual significance.

**Fig. 1:**
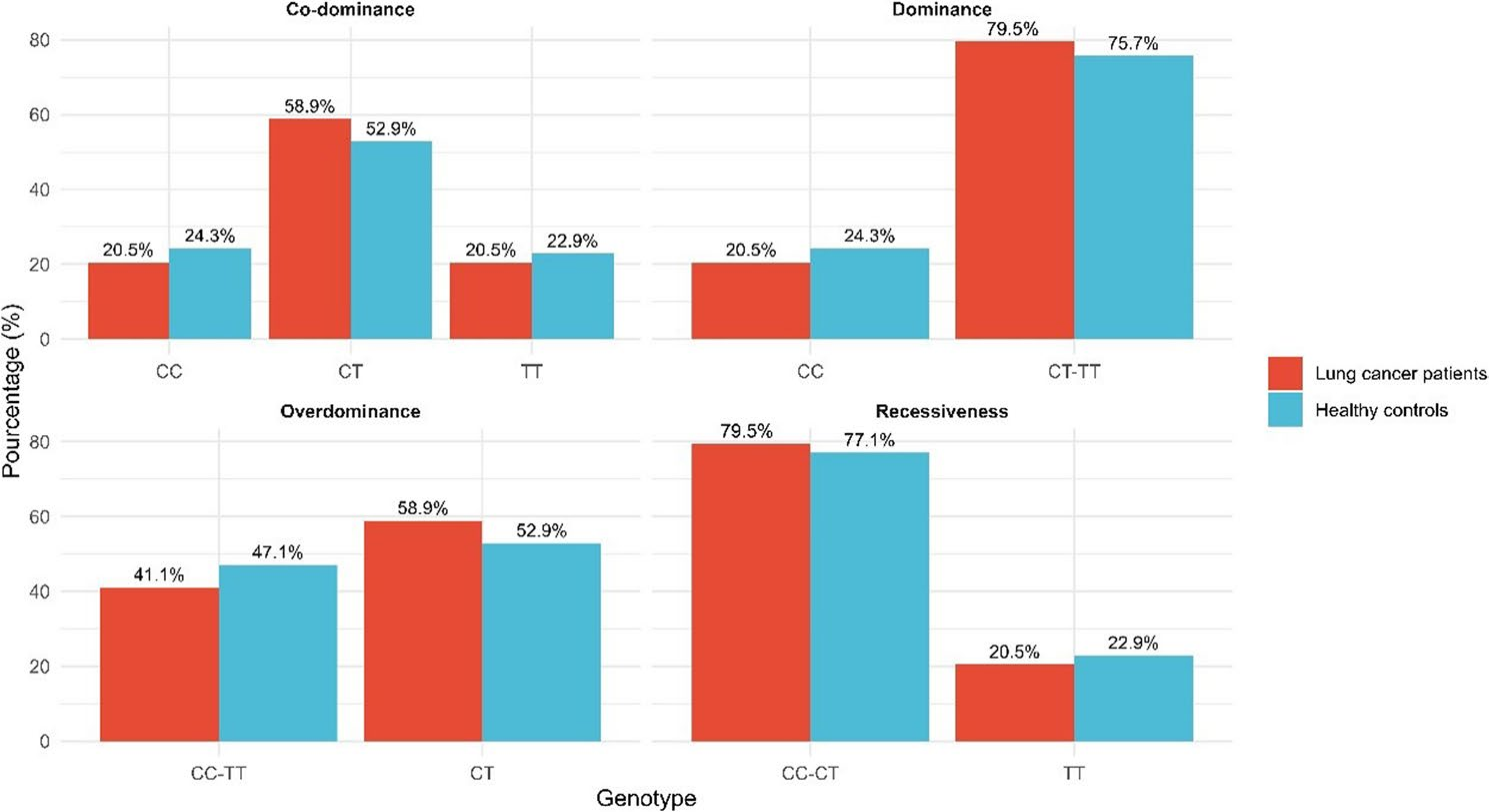
Distribution of rs2072454 *EGFR* SNP genotypes by genetic model. Comparison between lung cancer patients and healthy controls

**Fig. 2:**
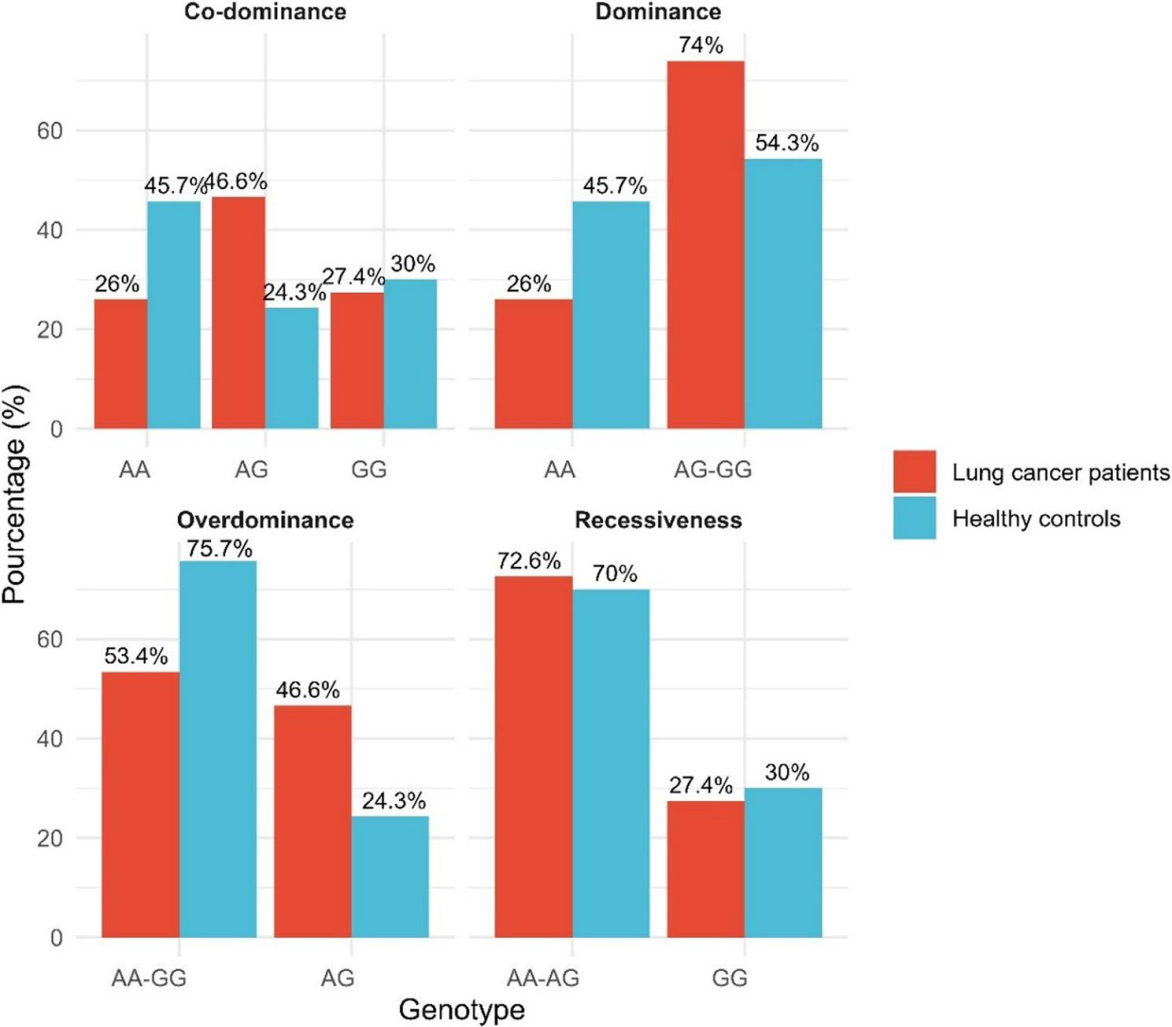
Distribution of rs2227983 *EGFR* SNP genotypes by genetic model. Comparison between lung cancer patients and healthy controls

While the TT genotype of rs2072454 and the GG genotype of rs2227983 both exhibited odds ratios greater than 1, their 95% confidence intervals included unity (*p* > 0.05), indicating these associations did not reach statistical significance. The genotype distributions of rs2072454 and rs2227983 SNPs across different genetic models are illustrated in Figs. 1 and 2 respectively.

### Genotypic and allelic frequencies of the two *EGFR* SNPs (rs2072454 and rs2227983) in both cases of adenocarcinoma and controls

**Fig. 3:**
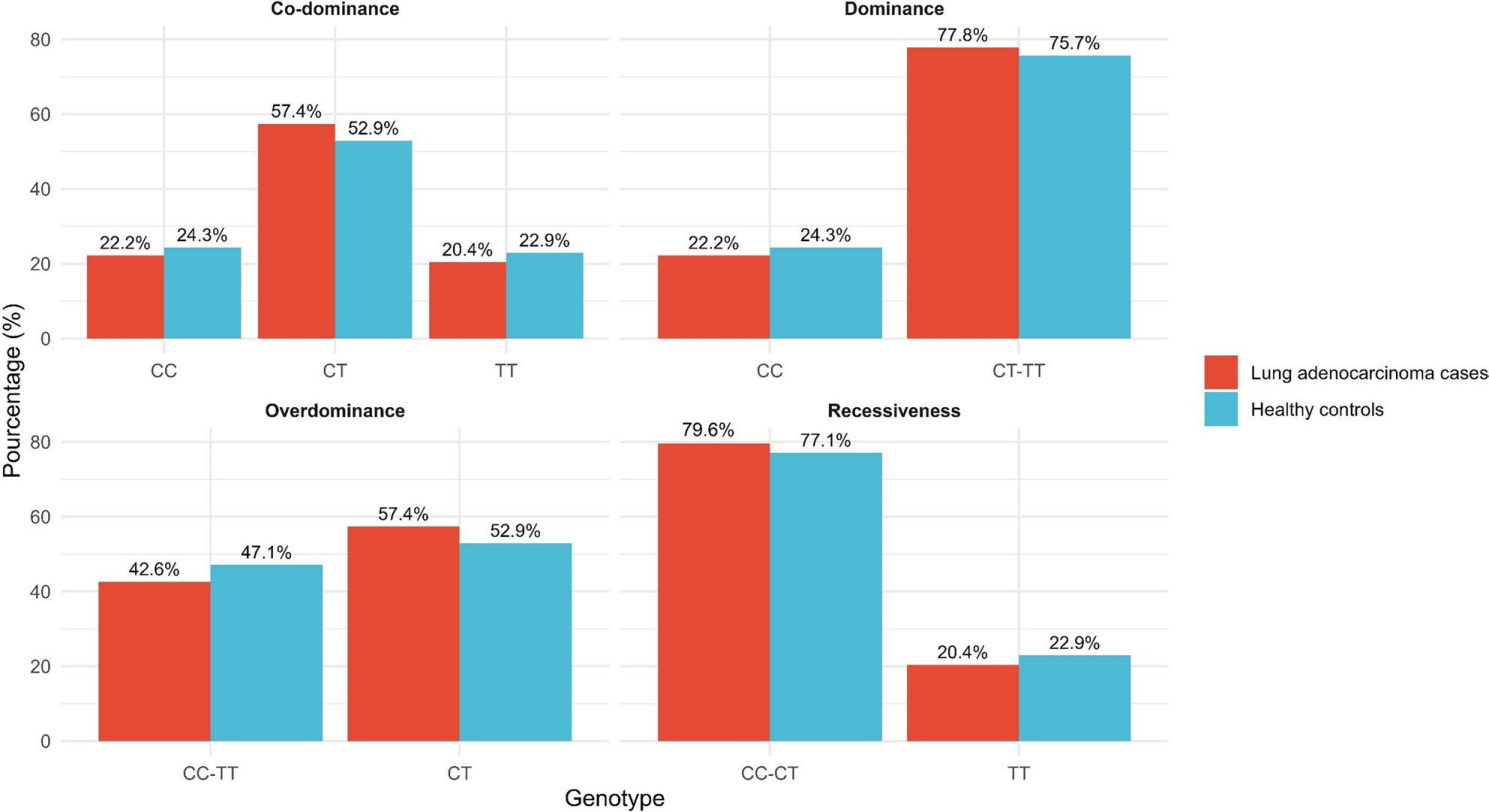
Distribution of rs2072454 *EGFR* SNP genotypes by genetic model. Comparison between lung adenocarcinoma patients and healthy controls

**Fig. 4:**
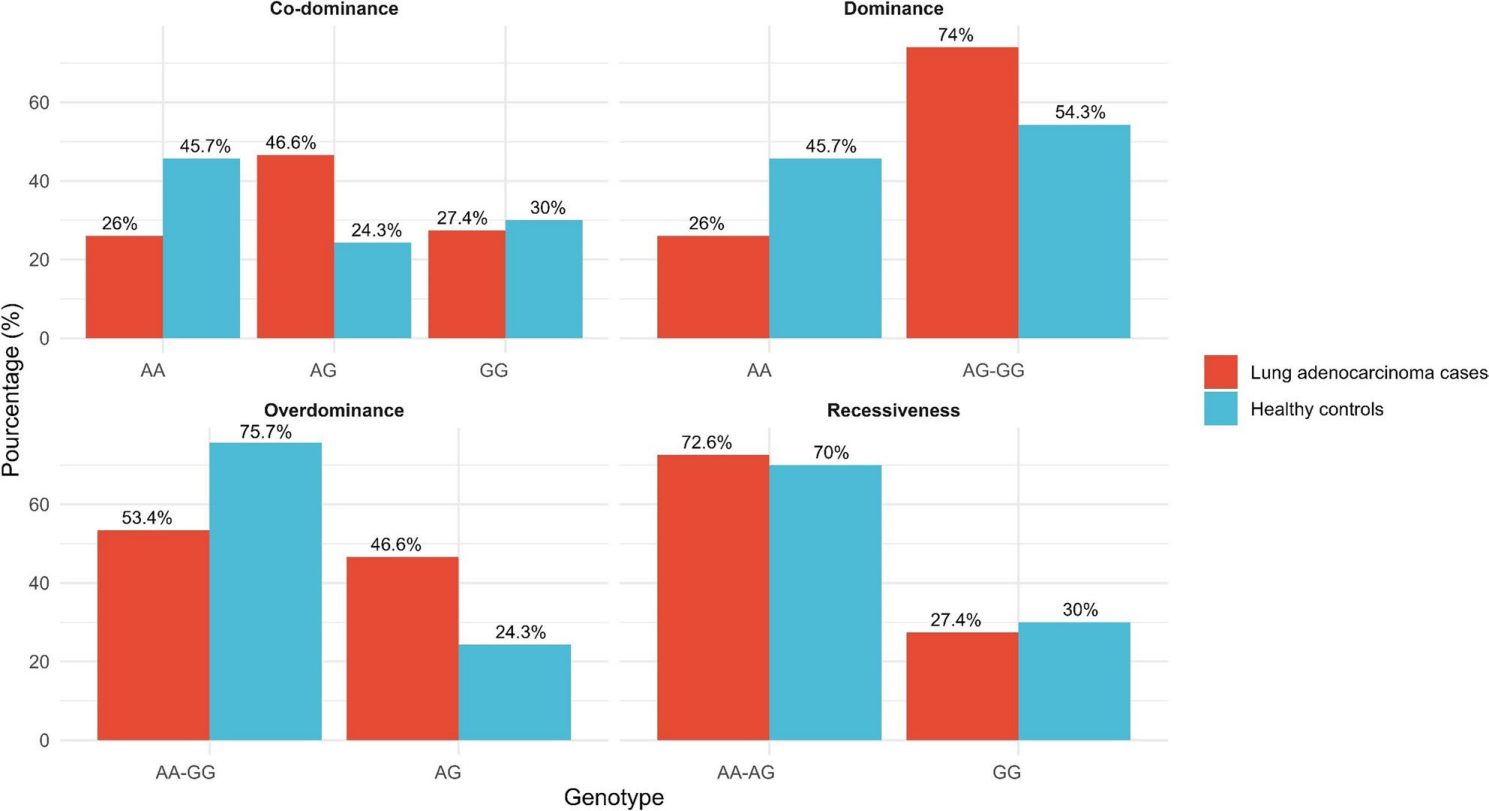
Distribution of rs2227983 *EGFR* SNP genotypes by genetic model. Comparison between lung adenocarcinoma patients and healthy controls

The frequency distribution of the *EGFR* SNPs in both lung adenocarcinoma subgroup and control group are shown in Table 1. The CT heterozygous genotype of the rs2072454 SNP was the most frequent in both cases and controls groups compared to the homozygous genotypes TT and CC. For the rs2227983, the AG heterozygous genotype was the most frequent in the lung adenocarcinoma patients’ group. Moreover, genetic model selection identified the dominant model (AG/GG vs AA) as optimal for rs2227983 (AIC = 142.5, BIC = 153.8). However, logistic regression under the co-dominant model revealed a nominally significant association for the AG genotype (OR = 2.76, 95% CI: 1.01–7.5, *p* = 0.04; AIC = 144.0, BIC = 158.1), while no significant association was observed under the dominant model (OR = 2.30, 95% CI: 0.95–5.6, *p* = 0.06). The 95% confidence intervals for both models included the null value (1.0), indicating uncertainty in the effect size estimates. The details regarding the distribution of genetic models for both SNPs (rs2072454 and rs2227983) are shown in Figs. 3 and 4, respectively.

### Haplotype analysis of the two *EGFR* SNPs (rs2072454 and rs2227983)

Haplotype analysis was performed in comparison with the control group, once with the lung cancer group and once with the adenocarcinoma subgroup exclusively. The Table 2 shows the frequency distribution of the four haplotypes of the two *EGFR* SNPs in comparison to the control group with both the lung cancer group and the adenocarcinoma subgroup. The OR obtained for the four haplotypes showed no significant association between these haplotypes and the risk of developing lung cancer or adenocarcinoma. Additionally, no evidence of linkage disequilibrium (LD) was identified between the two *EGFR* SNPs, as shown by comparisons with the lung cancer group (*p* = 0.63) or the adenocarcinoma group (*p* = 0.98).

**Table 2:** Frequency distribution of haplotypes in the control group compared to the lung cancer group and the lung adenocarcinoma subgroup

Haplotypes	rs2072454	rs2227983	Frequencies (LC vs. Controls) *N* = 143	*p*-val	Frequencies (ADC vs. Controls) *N* = 124	*p*-val
1	C	A	0.2789	-	0.2640	-
2	C	G	0.2246	0.85	0.2440	0.22
3	T	A	0.2560	0.86	0.2561	0.40
4	T	G	0.2404	0.37	0.2357	0.17

*LC* Lung Cancer, *ADC * Adenocarcinoma

## Discussion

This study represents, to our knowledge, the first investigation of EGFR rs2072454 and rs2227983 polymorphisms in relation to lung cancer risk in a Western Algerian population. While our primary analysis focused on genetic associations, the regression model confirmed smoking status as a strong predictor of lung cancer risk in this cohort, consistent with established epidemiological evidence estimating that almost 90% of lung cancer in men and 70 to 80% in women are due to cigarette smoking [12]. The marginal association for gender may reflect either limited power to detect sex-specific differences or genuine biological homogeneity in this population.

Importantly, in our analysis of *EGFR* polymorphisms (rs2072454 and rs2227983), we observed strikingly different risk patterns between overall lung cancer cases and ADC subgroups. When examining all histological subtypes, the heterozygous CT genotype of rs2072454 demonstrated a potential association with increased lung cancer risk under the overdominant model, while rs2227983 showed no significant association. 

In contrast, this pattern reversed noticeably when focusing specifically on ADC. In the ADC group, rs2072454 did not show a significant association, whereas rs2227983 emerged as a possible risk factor under the co-dominant model, with the AG genotype conferring a 2.76-fold increased risk, despite the dominant model (AG/GG vs. AA) was statistically optimal for rs2227983 per AIC/BIC criteria but showed no significant association under multivariable logistic regression evaluation. While we reported the AG genotype’s nominal significance, its absence in the dominant model necessitate validation. However, given the modest sample size and borderline statistical significance, this finding should be interpreted with caution. 

Meanwhile, even though no significant association was found under the dominant model in the rs2227983, this model was selected according to the AIC/BIC criteria, which could support the hypothesis of dominant-negative effects in heterozygotes if the AG genotype showed a significant association under this model [13]. As described in the literature, this SNP causes a non-synonymous change from arginine (R) to lysine (K) at position 521, reducing receptor activity and attenuating ligand binding and ligand-induced EGFR signalling [11]. As a result, individuals with the AG or GG genotype, who carry one or two copies of the G allele, may exhibit impaired EGFR signalling, which could promote tumorigenesis or reduce the effectiveness of normal growth control mechanisms. However, our analysis revealed a potential association between the AG heterozygote genotype and lung adenocarcinoma (ADC) susceptibility under the co-dominant model, suggesting a functional role for the variant allele (G) in *EGFR*-mediated oncogenesis. This could arise through either (1) independent action of both wild-type (A) and variant (G) EGFR proteins, leading to dysregulated signalling due to altered dimerization or ligand affinity, paralleling the PI MZ heterozygous phenotype in α1-antitrypsin deficiency where both protein forms contribute to pathology [14], or (2) a haploinsufficiency gain-of-function effect, where the G allele enhances proliferative activity while the A allele alone becomes insufficient for normal function [13, 15]. Such dose-dependent effects align with recent evidence that heterozygous oncogenic variants can promote tumorigenesis through quantitative changes in protein function, underscoring the importance of intermediate-risk alleles in cancer predisposition [15].

We acknowledge that these mechanistic interpretations remain speculative and require functional validation in future studies.

Additionally, rs2072454 is located within the 2 bases of an exon–intron junction of the *EGFR* gene and results in a synonymous change. It has been shown that although a synonymous change doesn’t result in a protein sequence change, it may affect its number, structure, activity and function by affecting selective splicing and translation kinetics [16, 17]. Indeed, it has been predicted that the rs2072454 SNP may affect mRNA splicing, which can lead to abnormal mRNA isoforms and, consequently, alter protein function or expression levels [8]. Thus, individuals carrying both C and T alleles may have a phenotypic advantage linked to an increased risk of lung cancer. Future studies are necessary to validate this hypothesis experimentally through the implementation of minigene splicing assays or RNA sequencing of patient-derived samples. This will help determine whether this SNP influences *EGFR* transcript processing and contributes to lung cancer susceptibility.

Haplotype analyses showed no significant association between the different haplotypes and lung cancer in general or adenocarcinoma. This finding suggests that the haplotypes formed by the studied *EGFR* SNPs do not contribute to lung cancer susceptibility. Furthermore, no evidence for linkage disequilibrium between the two *EGFR* SNPs was found when compared with both lung cancer and adenocarcinoma groups. This suggests that the alleles at these two loci segregate and act independently of each other with respect to their effect on lung cancer risk in the studied populations.

Our results align with other findings by Lawi et al. [18] that reported an association of the rs2227983 with an increased risk of NSCLC.

However, these results contrast with recent findings by Bashir et al. [6] where the TT genotype of the rs2072454 was related to a risk of lung adenocarcinoma and no evident association was found between the rs2227983 polymorphism and lung cancer or adenocarcinoma risk. Furthermore, Yingfu et al. [19] found that the rs2072454 CT-TT genotype was identified as a risk factor for NSCLC in a dominant model, and the AG-GG genotype at rs2227983 was associated with squamous cell carcinoma risk in a dominant mode. Moreover, a study by Choi et al. [8] reported no significant association between lung cancer risk and the rs2072454 polymorphism.

The observed differences in the association between the rs2072454 and rs2227983 polymorphisms and lung cancer risk across studies may be attributed to several factors. First, Ethnicity plays a significant role, as genetic backgrounds and allele frequencies can vary across populations, potentially influencing the strength or direction of associations observed in different studies [20, 21]. Additionally, the histological type of lung cancer is another important parameter, some studies specifically analysed NSCLC or its subtypes such as ADC or SqCC, while others may have included a broader range of histological types, which could dilute or obscure subtype-specific associations. Other potential reasons for these inconsistencies include differences in environmental exposures, such as smoking status, as well as variations in study design, genotyping methods, and adjustment for confounding factors, all of which can influence the observed associations between genetic polymorphisms and lung cancer risk. When considered as a whole, these parameters highlight the complex nature of genetic association studies in lung cancer. They also underscore the necessity of considering population characteristics, tumour histology, and environmental exposures when interpreting divergent results.

Additionally, other *EGFR* polymorphisms have been implicated in lung cancer susceptibility. For example, rs712829 (G allele) demonstrated consistent associations with increased risk across multiple genetic models in meta-analyses [22], while Liu et al. [23] reported that the TT genotype of rs6965469 and the AA genotype of rs763317 significantly elevated lung cancer risk. Another study from Zuo et al. [14] showed that *EGFR* rs2293347 was associated with lung adenocarcinoma, squamous cell carcinoma and small cell lung cancer susceptibility, and that *EGFR* rs884225 increases lung adenocarcinoma risk.

Moreover, a study from Pezeshki et al. [24] has demonstrated that *EGFR* rs712829 G allele was associated with a risk of lung cancer while the T allele and TT genotype showed a protective role against lung cancer.

The broader context of *EGFR* polymorphism research reveals complex patterns. Yingfu et al. [19] also identified rs1050171 as a risk-associated variant, expanding the list of potentially relevant *EGFR* SNPs. Interestingly, the AG genotype of the rs2227983 has been linked to mild/moderate allergic asthma in women [25], suggesting pleiotropic effects unrelated to oncogenesis. Similarly, the rs2072454 T allele and TT genotype were associated with gastric cancer risk [26]. Moreover, Mustapha et al. [11] showed that the rs2227983 was involved in both colorectal cancer susceptibility and response to therapeutics, highlighting tissue-specific roles for *EGFR* variants in carcinogenesis. Butkiewicz et al. [27] showed that in unresectable NSCLC patients treated with radiotherapy and platinum-based chemotherapy, the rs712830 CC genotype was associated with decreased overall survival, while the rs712829 TT genotype correlated with reduced locoregional-free survival.

## Limitations of the study

Our study has some limitations that should be considered, the lack of significant haplotype association with lung cancer risk may be due to the limited number of SNPs analysed. Specifically, our analysis was limited to only two SNPs, resulting in a simpler haplotype structure with fewer possible combinations and potentially less power to detect associations. In contrast, Bashir et al. [6] examined haplotypes composed of four SNPs (rs712829, rs712830, rs2072454, and rs11543848), which increases the genetic diversity and the likelihood of identifying haplotypes that may be associated with disease risk. Studies have shown that the combined effect of multiple SNPs can reveal associations that are not apparent when analysing individual SNPs or simpler haplotypes, as the interaction between several genetic variants may better capture the underlying genetic architecture of lung cancer [28, 29]. In addition, the unmatched distribution of gender and smoking status may have introduced confounding bias. We adjusted for these variables in multivariate analyses. However, residual confounding could persist due to unmeasured interactions, such as gender-specific smoking behaviours or differential genetic susceptibility. Future case-control studies with matched recruitment would help validate the robustness of our results.

Given the exploratory design of this study, post-hoc power calculations were not performed, as they would not add meaningful information beyond the reported confidence intervals and p values [30].

## Conclusions

In summary, our findings highlight that heterozygous CT genotype of the rs2072454 *EGFR* SNP is potentially associated with increased lung cancer risk, while the heterozygous AG genotype of the rs2227983 *EGFR* SNP showed a borderline association with increased lung ADC risk.

Despite the exploratory nature of this study, its limited sample size and the disparity in smoking status and gender, our findings provide new insights into the relationship between *EGFR* rs2072454 and rs2227983 polymorphisms and lung cancer risk, specifically in an Algerian population, contribute to the understanding of genetic susceptibility in diverse ethnic groups and may inform personalized risk assessment in lung cancer management.

Moreover, these results underscore the multifactorial nature of *EGFR*’s role in lung cancer, influenced by factors such as genetic background, tumour histology, and environmental exposure. The observed variability across studies further emphasizes the importance of considering population diversity and the inclusion of a broader range of genetic variants in future research. Expanding haplotype analyses to include additional *EGFR* SNPs and conducting studies in larger, ethnically diverse cohorts may help clarify the genetic determinants of lung cancer risk and improve our understanding of *EGFR*’s contribution to carcinogenesis. Prospective cohorts with balanced smoking with stratified samples and functional assays, such as minigene splicing for rs2072454 are needed to validate these findings.

Ultimately, these efforts could inform the development of more precise genetic models related to the risk of developing lung cancer, predict the treatment response and provide targeted prevention and screening strategies for lung cancer.

## Data Availability

The datasets used and/or analysed during the current study are available from the corresponding author on reasonable request.
